# Comparative Transcriptomic Analysis to Identify the Important Coding and Non-coding RNAs Involved in the Pathogenesis of Pterygium

**DOI:** 10.3389/fgene.2021.646550

**Published:** 2021-03-15

**Authors:** Xin Liu, Jing Zhang, Danyao Nie, Kun Zeng, Huiling Hu, Jinjun Tie, Liangnan Sun, Ling Peng, Xinhua Liu, Jiantao Wang

**Affiliations:** ^1^Shenzhen Eye Hospital, Shenzhen Key Laboratory of Ophthalmology, Affiliated Shenzhen Eye Hospital of Jinan University, Shenzhen, China; ^2^Department of Ophthalmology, The Affiliated Hospital of Guizhou Medical University, Guiyang, China

**Keywords:** messenger RNA, long non-coding RNA, circular RNA, RNA-seq, pterygium

## Abstract

Pterygium is a common ocular surface disease characterized by abnormal fibrovascular proliferation and invasion, similar to tumorigenesis. The formation of tumors is related to a change in the expression of various RNAs; however, whether they are involved in the formation and development of pterygium remains unclear. In this study, transcriptome analysis of messenger RNAs (mRNAs), long non-coding RNAs (lncRNAs), and circular RNAs (circRNAs) of paired pterygium and normal conjunctiva was performed to explore key genes regulating the development of pterygium. In total, 579 mRNAs, 275 lncRNAs, and 21 circRNAs were differentially expressed (DE) in pterygium compared with paired conjunctival tissues. Functional enrichment analysis indicated that DE RNAs were associated with extracellular matrix organization, blood vessel morphogenesis, and focal adhesion. Furthermore, through protein-protein interaction network and mRNA-lncRNA co-expression network analysis, key mRNAs including *FN1*, *VCAM1*, and *MMP2*, and key lncRNAs including *MIR4435-2HG* and *LINC00968* were screened and might be involved in the pathogenesis of pterygium. In addition, several circRNAs including *hsa_circ_0007482 and hsa_circ_001730* were considered to be involved in the pterygium development. This study provides a scientific basis for elucidating the pathogenesis of pterygium and will be beneficial for the development of preventive and therapeutic strategies.

## Introduction

Pterygium is a common ocular surface disease characterized by benign subconjunctival fibrous connective tissue hyperplasia and vascular invasion into the limbus, causing eye dryness, foreign body sensation, and even visual loss. Presently, there is no effective drug for the treatment of this disease, and surgery is the best treatment option; however, recurrence is common. Repeated recurrence may cause symblepharon adhesion and affect eye movement. Most researchers have reported that the pathogenesis of pterygium is related to an exposure to UV radiation, and occurrence of anti-apoptosis mechanisms, extracellular matrix remodeling, and other mechanisms, but the exact cause of pterygium remains unclear (Liu et al., 2017b). Pathologically, pterygium is characterized by proliferation, fibrosis, corneal infiltration, and angiogenesis, which are similar to those observed in tumor tissues (Chui et al., 2008). Epithelial-mesenchymal transition (EMT), a recent focus in the study of tumor pathogenesis, was reported to be involved in pterygium generation (Kato et al., 2007; Meshkani et al., 2019). The expression levels of EMT markers, such as *FN1* and *VIM*, were significantly higher in pterygium than those in normal conjunctiva (Engelsvold et al., 2013; Hou et al., 2014). Additionally, angiogenesis, an important pathological mechanism of disease development, participates in the occurrence and development of pterygium (Livezeanu et al., 2011). The formation of pterygium is extremely complex, and its complete pathogenesis remains to be further explored.

In recent years, non-coding RNAs, including long non-coding RNAs (lncRNAs) and circular RNAs (circRNAs), have been reported to play crucial roles in transcriptional regulation of gene expression and post-transcriptional levels (Jiang et al., 2015; Gao et al., 2017). lncRNAs are autonomously transcribed non-coding RNAs greater than 200 nt in length (Wilusz et al., 2009). circRNAs are widespread endogenous non-coding RNAs that forms covalently bonded closed-loop structures due to the absence of 5' and 3' ends (Hao et al., 2020). Non-coding RNAs are involved in several biological processes, including cell proliferation, differentiation, and apoptosis, and their abnormal expression is directly related to the occurrence of many diseases, such as cancer and retinitis pigmentosa (Beermann et al., 2016; Donato et al., 2020c). Multiple lncRNAs and circRNAs have been found to be differentially expressed (DE) in pterygium compared with conjunctival tissues *via* microarray analysis (Liu et al., 2016; Li et al., 2018a). Thus far, the data available on pterygium are insufficient, and the complete profile and roles of coding and non-coding RNAs in the pathogenesis of pterygium remain unknown. In this study, we compared the expression patterns of coding and non-coding RNAs in pterygium tissues and paired normal conjunctival tissues by RNA-seq to clarify the pathogenesis of pterygium, and to provide new clues for the development of strategies for its prevention and treatment, and to reduce postoperative recurrence.

## Materials and Methods

### Sample Preparation From Patients

The samples used in this study were from six patients with primary pterygium from the Shenzhen Eye Hospital. All patients were in good health, aged between 55 and 70 years, and had undergone pterygium excision combined with conjunctival autograft. Pterygium tissue was collected from the nasal limbus, and a small piece of conjunctival tissue was collected as a control. Pterygium tissues and paired normal conjunctival tissues collected from three patients were placed in normal saline to wash the residual blood on the surface, and then quickly placed in liquid nitrogen for rapid freezing, and finally stored at −80°C for total RNA extraction. In addition, samples from the other three patients were fixed in 4% paraformaldehyde (PFA) and stored at 4°C for histological examination. This study was approved by the Ethics Committee of the Shenzhen Eye Hospital. All patients signed an informed consent form and voluntarily participated in the study.

### Immunohistochemical Analysis

Three pairs of paraffin-embedded pterygium tissues and normal conjunctival tissues were prepared for immunohistochemistry (IHC) studies according to the manufacturer’s instructions of the SignalStain®DAB Substrate Kit (Cell Signaling Technology, Beverly, MA, United States). The primary antibody used was rabbit anti-human CD31 (Abcam, Cambridge, United Kingdom) and the secondary antibody used was Alexa fluor 488-conjugated goat anti-rabbit IgG (Thermo Fisher Scientific, Waltham, MA, United States). The percentage of CD31-positive cells was calculated using the Image Proplus 6.0 software.

### RNA Extraction, Library Construction, and RNA-Seq

Total RNAs were extracted from three paired pterygiums and normal conjunctivas using TRIzol reagent, respectively (Invitrogen, Carlsbad, CA, United States). RNA degradation and contamination were monitored on 1% agarose gels, and RNA purity was checked using the NanoPhotometer® spectrophotometer (Implen, Westlake Village, CA, United States). Additionally, RNA concentration and integrity were determined using a Qubit® RNA Assay Kit with a Qubit® 2.0 Fluorometer (Life Technologies, Carlsbad, CA, United States) and an RNA Nano 6,000 Assay Kit with the Bioanalyzer 2,100 system (Agilent Technologies, Santa Clara, CA, United States), respectively. Ribosomal RNAs from each sample were removed using Ribo-Zero Gold Kits (Epicenter, Madison, WI, United States). Six chain-specific libraries were constructed using NEBNext® Ultra™ RNA Library Prep Kit for Illumina® (NEB, Ispawich, MA, United States) following the manufacturer’s recommendations, and Qubit 2.0 and quantitative real-time PCR (qRT-PCR) were used to check the quality of the library. Finally, RNA-seq was performed using an Illumina NovaSeq 6,000 instrument (Illumina, San Diego, CA, United States). The raw data obtained had been submitted to the NCBI database (accession number: PRJNA669964).

### Reference Genome Mapping and Transcriptome Assembly

To obtain clean reads, low quality reads and reads containing adapter contamination or ploy-N, were removed. Simultaneously, the Q20, Q30, and GC contents of the clean data were calculated to assess their quality. All downstream analyses were based on high-quality clean data. The software HiSAT2 was used to map the sequence data to the human reference genome (GRCh38; Kim et al., 2015).

### Identification of lncRNAs and circRNAs

Based on the mapped results, transcriptome assembly and reconstruction were performed using StringTie software (Pertea et al., 2015). Candidate lncRNAs were identified by the following workflow: (1) Transcripts with exons ≥2 and lengths >200 bp were obtained; (2) the known transcripts including mRNAs and other non-coding RNAs were filtered by comparing with annotation files using Cuffcompare (Trapnell et al., 2010); and (3) coding potential analysis were performed by coding potential calculator (CPC), coding-non-coding index (CNCI), and Pfam (Sonnhammer et al., 1998; Kong et al., 2007; Sun et al., 2013). Finally, the remaining transcripts with no coding potential were defined as candidate novel lncRNAs. To improve the accuracy of circRNA identification, a conjoint analysis of the two software, CIRI and find_circ, was used with default parameters (Sekar et al., 2019).

### Principal Component Analysis

Principal component analysis (PCA) is performed to obtain an overview of the expression profile of mRNAs, lncRNAs, and circRNAs using OmicShare tools.^1^

### Analysis of Differentially Expressed mRNAs, lncRNAs, and circRNAs

The fragments per kilobase of transcript per million reads mapped (FPKM) value was used to determine the expression levels of mRNAs and lncRNAs, while the expression levels of circRNAs in each sample were calculated using transcripts per kilobase million mapped (TPM; Mandric et al., 2017). Significance analysis of the two groups was performed using the DEseq2 R package (Love et al., 2014), and an FDR-adjusted *p* < 0.05 and log2 fold change > 1.0 were used to screen for DE genes.

### Functional Analysis of mRNAs, lncRNAs, and circRNAs

The function of the DE lncRNAs was predicted according to the co-location and co-expression correlation of lncRNAs and mRNAs. For determining the mechanism of cis regulation, we set the co-location threshold to 100 kb upstream and downstream of lncRNA. For determining the mechanism of trans-regulation, Pearson’s correlation coefficient ≥ 0.9 was defined as the target correlation. The function of DE circRNAs was revealed *via* functional analysis of their parental genes. Gene ontology (GO) functional enrichment analysis and Kyoto Encyclopedia of Genes and Genomes (KEGG) pathway analysis were performed using the clusterProfiler R package (Yu et al., 2012). The GO terms and KEGG pathways with *p* < 0.05 were indicated to be significantly enriched, and the top 15 enriched terms are shown in the figures.

### Protein-Protein Interaction Network Construction

To investigate the interactive relationships of DE mRNAs, a protein-protein interaction (PPI) network was constructed using the STRING database^2^ with a combined score >0.4 as the cutoff criterion. PPI network visualization was carried out using Cytoscape software (version 3.5.1).

### Co-expression Network Construction

A co-expression network of DE lncRNAs with their target-associated DE mRNAs was constructed using Cytoscape software (version 3.5.1) to explore the function of key lncRNAs.

### Gene Expression Validation by Quantitative Real-Time PCR

To verify the sequence of circRNAs, PCR reactions were performed using Takara Ex Taq polymerase (Takara, Dalian, China) following the manufacturer’s instructions. PCR products of the circRNAs were detected by conducting 2% agarose gel electrophoresis, and Sanger sequencing was performed at TSINGKE Biotechnology Co., Ltd. (Beijing, China). In the RNase R digestion assay, the RNAs extracted from pterygium and normal conjunctiva were divided into two parts: one part was incubated with RNase R (Geneseed, Guangzhou, China) for 30 min, and the expression levels of linear RNA and circular RNA were detected by qRT-PCR; for the other part, the RNAs were directly used for the detection of the expression of linear RNA and circular RNA by qRT-PCR (control group, Mock).

Gene expression levels were verified using qRT-PCR. First-strand cDNA was synthesized from three pairs of total RNA samples from pterygium and conjunctive groups using the PrimeScript™ Master mix (Takara), following the manufacturer’s instructions. qRT-PCR was performed using SYBR Premix Ex Taq™ (Takara) and the Step-OnePlus™ Real-time PCR System (Applied Biosystems, Foster City, CA, United States). The reaction conditions were as follows: 95°C for 30 s, followed by 40 cycles of 95°C for 5 s and 60°C for 30 s. Each qRT-PCR assay was performed in triplicate, and glyceraldehyde-3-phosphate dehydrogenase (GAPDH) gene was used as the normalization control. All primers used in the qRT-PCR are shown in Supplementary Table S1. The relative gene expression level was calculated using the 2^−*Δ*ΔCT^ method.

### Statistical Analysis

Experiments were performed independently at least three times, and each independent test was conducted using three biological replicates. Statistical analyses of the data from CD31 immunohistochemistry and qRT-PCR were performed using GraphPad Prism 6.0. The Student’s *t*-test was used to assess the differences between pterygium and normal conjunctival groups. All data are expressed as mean ± standard deviation. Values of *p* < 0.05 were considered statistically significant.

## Results

### Differences in Morphology Between Pterygium and Normal Conjunctiva

**Figure 1:**
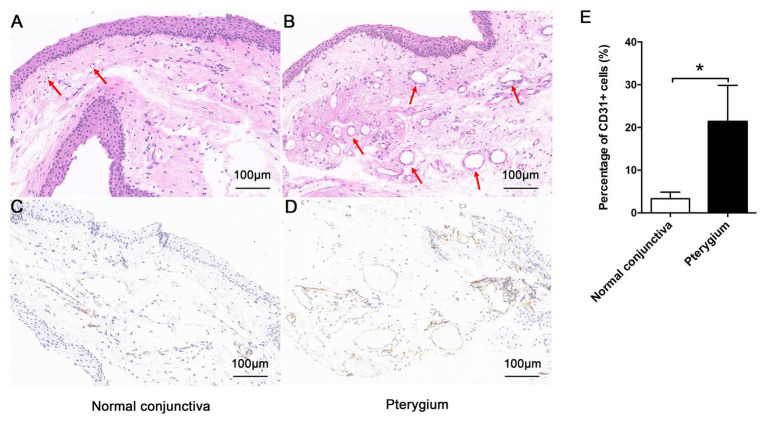
Morphologic characteristics of pterygium and normal conjunctiva. Morphology of normal conjunctiva **(A)** and pterygium **(B)** were observed by hematoxylin-eosin staining (×200), and the lumen has been indicated using a red arrow. The positive expression of CD31 in normal conjunctiva **(C)** and pterygium **(D)** was detected by immunohistochemistry (×200). **(E)** Percentage of CD31-positive cells in normal conjunctiva and pterygium were calculated. Data are represented as means ± SDs, *n* = 3 per group. ^*^p < 0.05.

As shown in Figures 1A,B, compared with normal conjunctiva, pterygium was richer in blood vessels and connective tissues. The positive expression of CD31 in pterygium connective tissue was significantly higher than that in normal conjunctiva, according to CD31 immunohistochemical staining results (Figures 1C–E).

### Summary of Raw Sequence Data

In this study, 657,824,860 clean reads were retained for the conjunctival and pterygium tissues after excluding low-quality reads. The average Q30 and GC content of the clean data were 93.60 and 48.04%, respectively, and more than 95.72% of the clean reads were mapped to the human reference genome (GRCh38; Table 1).

**Table 1:** Summary of sequencing data of different samples.

Sample_name	Raw_reads	Clean_reads	Q30 (%)	GC_content (%)	Total mapped	Mapped rate (%)
Conj_1	105,488,508	102,504,542	92.71	48.00	982,106,48	95.81
Conj_2	107,739,354	104,649,002	94.08	48.91	100,566,993	96.10
Conj_3	117,417,174	113,992,280	93.48	49.22	109,112,184	95.72
Pter_1	103,536,638	101,251,888	94.14	47.68	98,088,460	96.88
Pter_2	109,989,850	107,615,990	93.36	47.26	103,633,678	96.30
Pter_3	130,946,518	127,811,158	93.84	47.19	123,499,893	96.63

#### Expression Pattern of mRNA, lncRNA, and circRNA

After gene mapping, 19,775 mRNAs, 106,231 lncRNAs (including 2,884 novel lncRNAs), and 7,878 circRNAs (including 3,063 novel circRNAs) were identified in the conjunctiva and pterygium (Supplementary Table S2). Based on the matrix analysis by PCA, the expression profiles of all obtained mRNAs, lncRNAs, and circRNAs in this study could clearly distinguished pterygium from conjunctival tissue (Supplementary Figure S1). Furthermore, the first two components can explain 54.6, 44.3, and 44.3% variance of the mRNA, lncRNA, and circRNA dataset, respectively.

### DE Profiles of mRNAs, lncRNAs, and circRNAs

**Figure 2:**
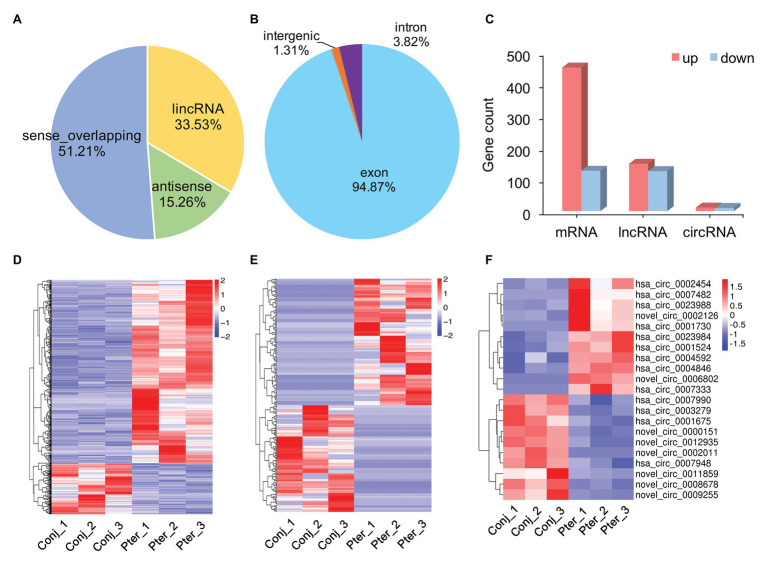
Gene expression characterization. **(A)** The type and proportion of novel long non-coding RNAs (lncRNAs) and **(B)** circular RNAs (circRNAs). **(C)** The count of differentially expressed (DE) messenger RNAs (mRNAs), lncRNAs, and circRNAs between pterygium and normal conjunctiva. Hierarchical clustering analysis of DE mRNAs **(D)**, lncRNAs **(E)**, and circRNAs **(F)** in pterygium and normal conjunctiva.

The novel lncRNAs were classified into four types, with the maximum proportion being sense_overlapping lncRNAs (Figure 2A). There were three types of circRNAs, of which exon circRNAs accounted for 94.87%, followed by intron circRNAs which counted for 3.82% (Figure 2B). Compared with conjunctival tissue, 579 mRNAs, 275 lncRNAs, and 21 circRNAs were differentially expressed in pterygium (Supplementary Table S3). Among them, 452 mRNAs, 149 lncRNAs, and 11 circRNAs were upregulated, and 127 mRNAs, 126 lncRNAs, and 10 circRNAs were downregulated in pterygium (Figure 2C). Cluster analysis showed that more than 75% of the DE mRNAs were upregulated, and nearly 50% of the DE lncRNAs and circRNAs were downregulated (Figures 2D–F).

### Functional Annotation of DE mRNAs, lncRNAs, and circRNAs

**Figure 3:**
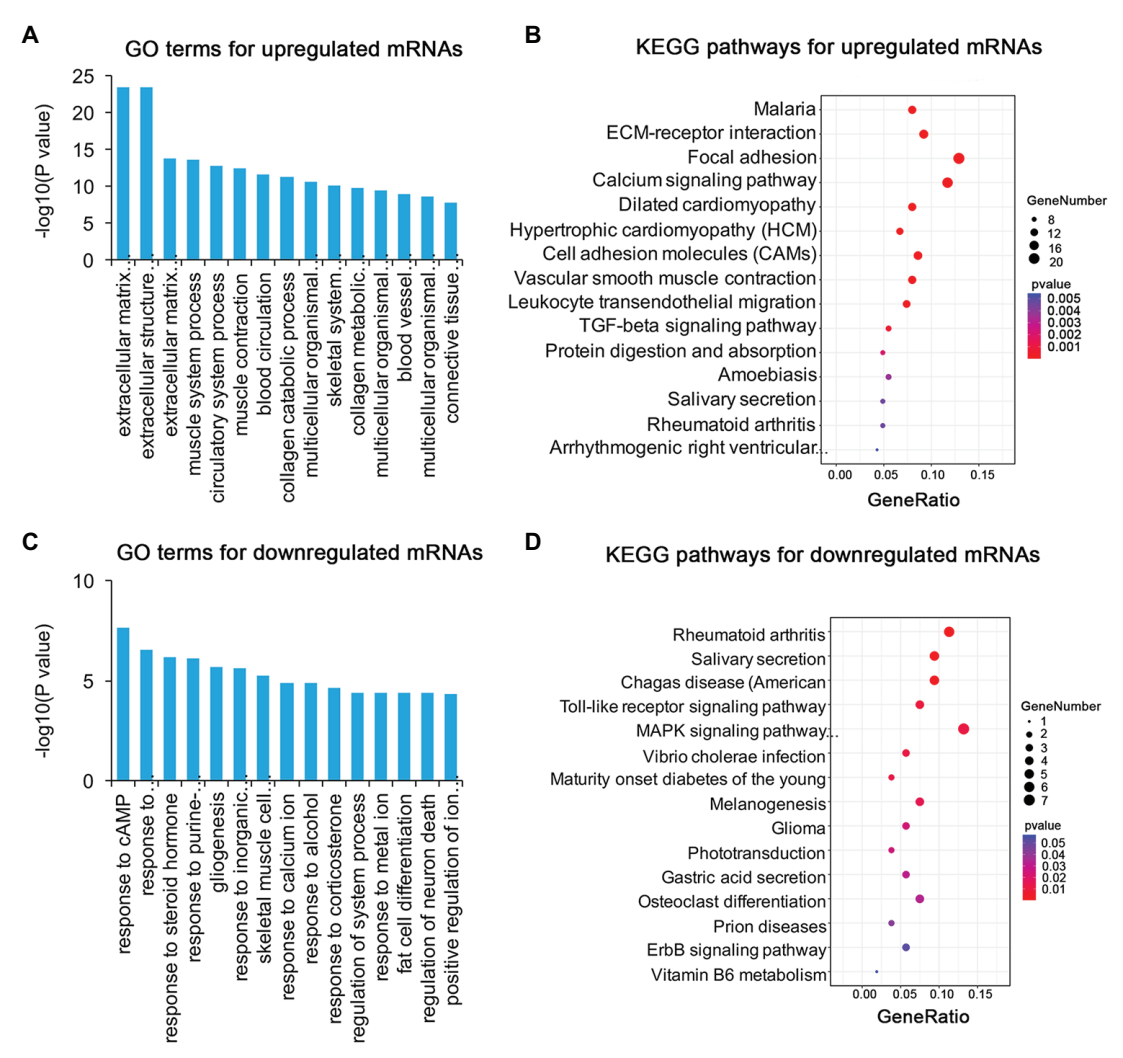
Gene ontology (GO) and Kyoto Encyclopedia of Genes and Genomes (KEGG) enrichment analysis of DE mRNAs in pterygium vs. normal conjunctiva. **(A,B)** The top 15 enriched biological processes and KEGG pathways for upregulated mRNAs. **(C,D)** The top 15 enriched biological processes and KEGG pathways for downregulated mRNAs.

**Figure 4:**
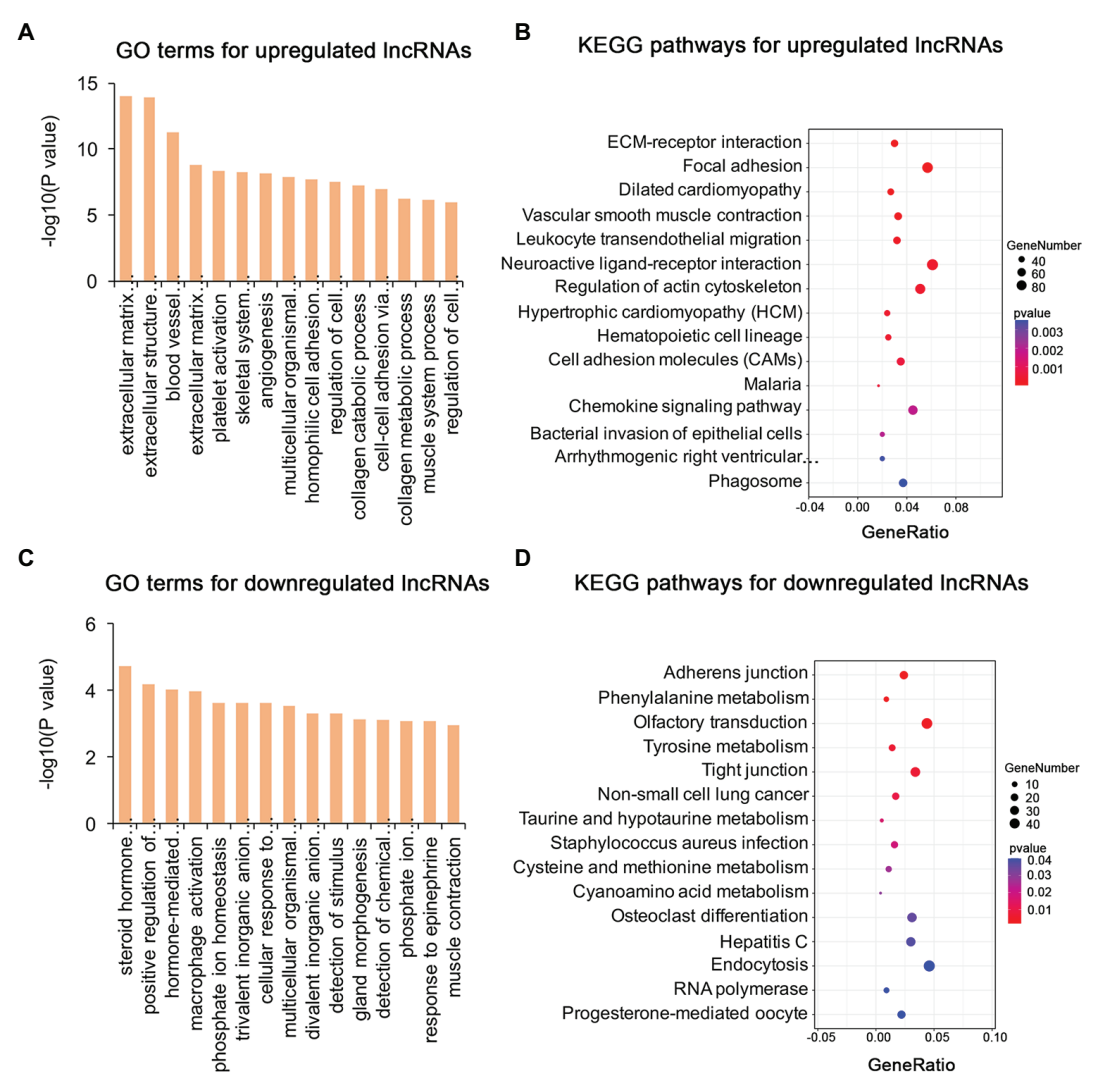
Gene ontology and KEGG enrichment analysis of target genes of DE lncRNAs in pterygium vs. normal conjunctiva. **(A,B)** The top 15 enriched biological processes and KEGG pathways for target genes of upregulated lncRNAs. (**C,D**) The top 15 enriched biological processes and KEGG pathways for target genes of downregulated lncRNAs.

**Figure 5:**
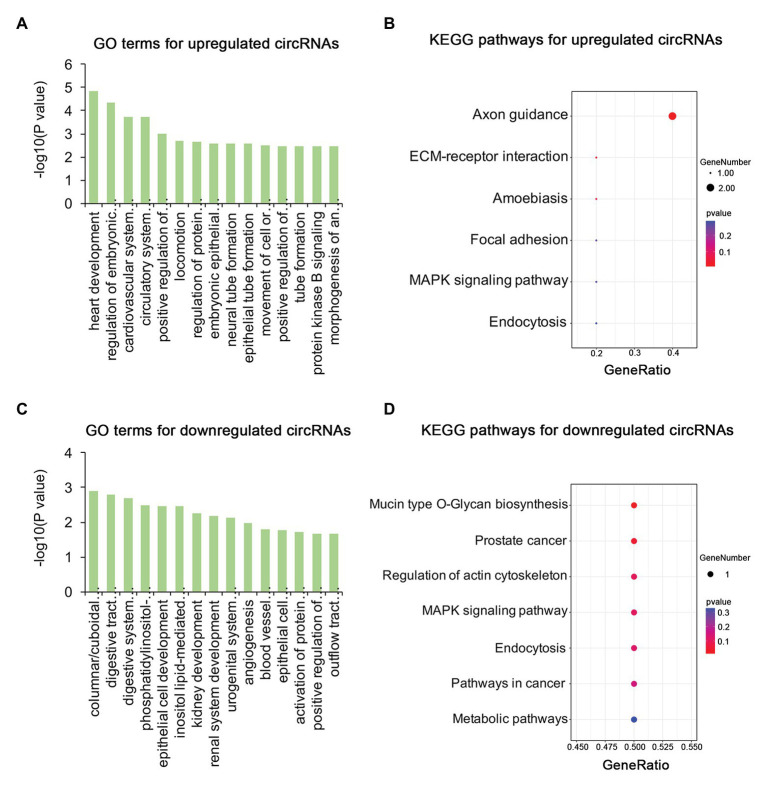
Gene ontology and KEGG enrichment analysis of parental genes of DE circRNAs in pterygium vs. normal conjunctiva. **(A,B)** The top 15 enriched biological processes and KEGG pathways for parental genes of upregulated circRNAs. **(C,D)** The top 15 enriched biological processes and KEGG pathways for parental genes of downregulated circRNAs.

To identify the mechanism of pterygium formation, GO functional enrichment analysis and KEGG pathway analysis of DE genes were conducted. As shown in Figure 3A; Supplementary Table S4, the significantly enriched GO terms for upregulated mRNAs were mainly associated with extracellular matrix organization, muscle system process, and blood vessel morphogenesis, while response to cAMP, response to calcium ion, and regulation of neuron death were the main enriched terms for downregulated mRNAs (Figure 3C; Supplementary Table S4). Similarly, the upregulated lncRNAs were mainly involved in extracellular matrix organization, blood vessel morphogenesis, and homophilic cell adhesion *via* plasma membrane adhesion (Figure 4A; Supplementary Table S4), while the downregulated lncRNAs were mainly related to positive regulation of extrinsic apoptotic signaling pathway *via* death domain receptors, hormone-mediated signaling pathway, and phosphate ion homeostasis (Figure 4C; Supplementary Table S4). Furthermore, the significantly enriched GO terms for upregulated circRNAs were mainly centered on positive regulation of protein kinase B signaling, epithelial tube formation, and positive regulation of apoptotic process (Figure 5A; Supplementary Table S4), while downregulated circRNAs were mainly involved in columnar/cuboidal epithelial cell differentiation, phosphatidylinositol-mediated signaling, and angiogenesis (Figure 5C; Supplementary Table S4). KEGG pathway analysis indicated that the upregulated mRNAs and lncRNAs were mainly involved in ECM-receptor interaction, focal adhesion, and vascular smooth muscle contraction (Figures 3B, 4B; Supplementary Table S5), while downregulated mRNAs were mainly enriched in Toll-like receptor signaling pathway, MAPK signaling pathway, and melanogenesis (Figure 3D; Supplementary Table S5); downregulated lncRNAs were related to adherens junction, phenylalanine metabolism, and tyrosine metabolism (Figure 4D; Supplementary Table S5). Furthermore, upregulated circRNAs were related to axon guidance (Figure 5B; Supplementary Table S5), and downregulated circRNAs were associated with mucin type O-Glycan biosynthesis (Figure 5D; Supplementary Table S5).

### PPI Network Analysis of DE mRNAs

**Figure 6:**
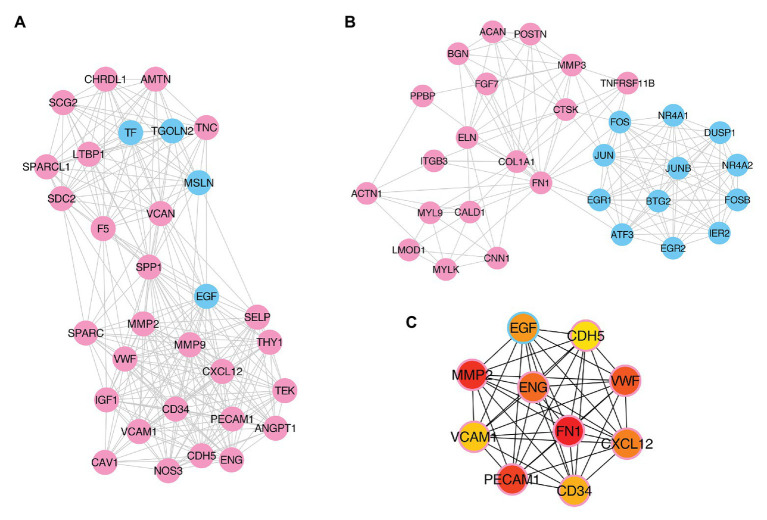
Identification of key modules and hub genes. **(A,B)** Two key modules identified by MCODE in Cytoscape. **(C)** Ten hub genes identified by CytoHubba in Cytoscape. Pink- and blue-colored terms represent up and downregulated mRNAs, respectively.

To further explore the pathogenesis of pterygium and to identify the key regulatory factors, the 579 DE genes were submitted to the STRING online database to analyze their protein interactions. The results showed that 489 DE mRNAs established interactions with each other, forming 2,528 edges, with a combined score >0.4 (Supplementary Table S5). Of these, the two most significant modules were detected using MCODE in Cytoscape. Module 1 was composed of 31 nodes and 252 edges (Figure 6A), while module 2 contained 30 nodes and 142 edges (Figure 6B). Furthermore, the top 10 hub genes, including *FN1*, *MMP2*, *PECAM1*, *VWF*, *ENG*, *CXCL12*, *EGF*, *CD34*, *VCAM1*, and *CDH5*, were identified using the cytoHubba plugin in the Cytoscape software by MCC (Figure 6C).

### Co-expression Network of mRNA-lncRNA

**Figure 7:**
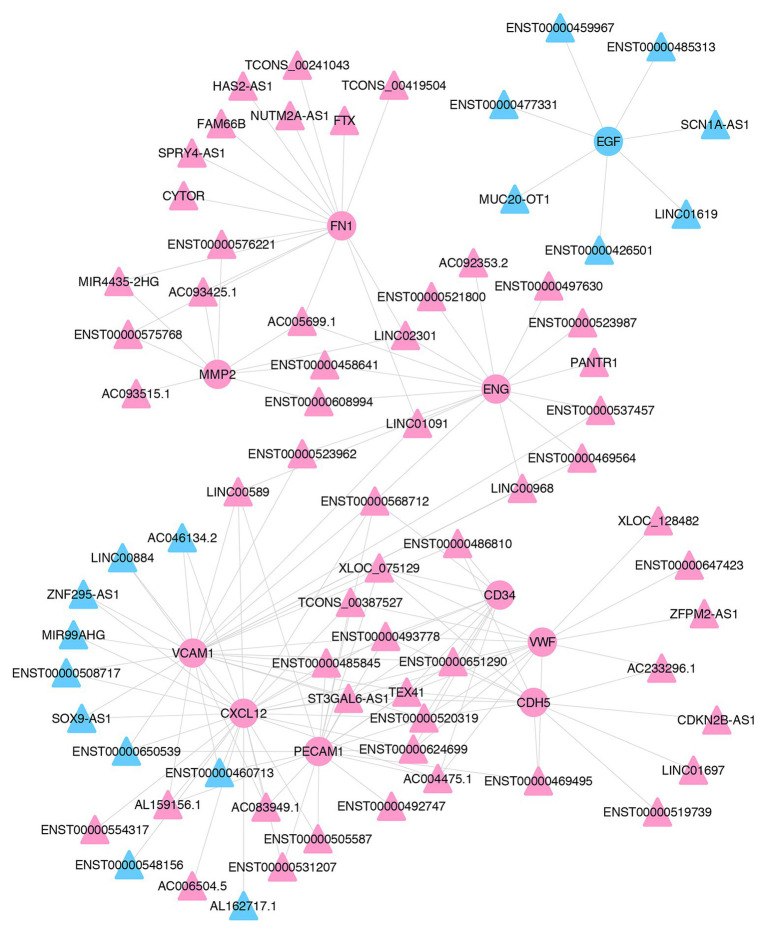
The co-expression network of lncRNAs and mRNAs. The co-expression network of 10 hub mRNAs and their trans-acting lncRNAs. Pink- and blue-colored terms represent up and downregulated genes, respectively. Circle and triangles represent mRNAs and lncRNAs, respectively.

To identify the key lncRNAs related to pterygium formation, 10 hub mRNAs and 72 target-associated DE lncRNAs were screened to construct an mRNA-lncRNA co-expression network using Cytoscape. As shown in the network, one lncRNA was associated with multiple mRNAs. For instance, *MIR4435-2HG* and *CDKN2B-AS1* were co-expressed with six mRNAs and three mRNAs, respectively (Figure 7; Supplementary Table S6).

### Validation of DE Genes

**Figure 8:**
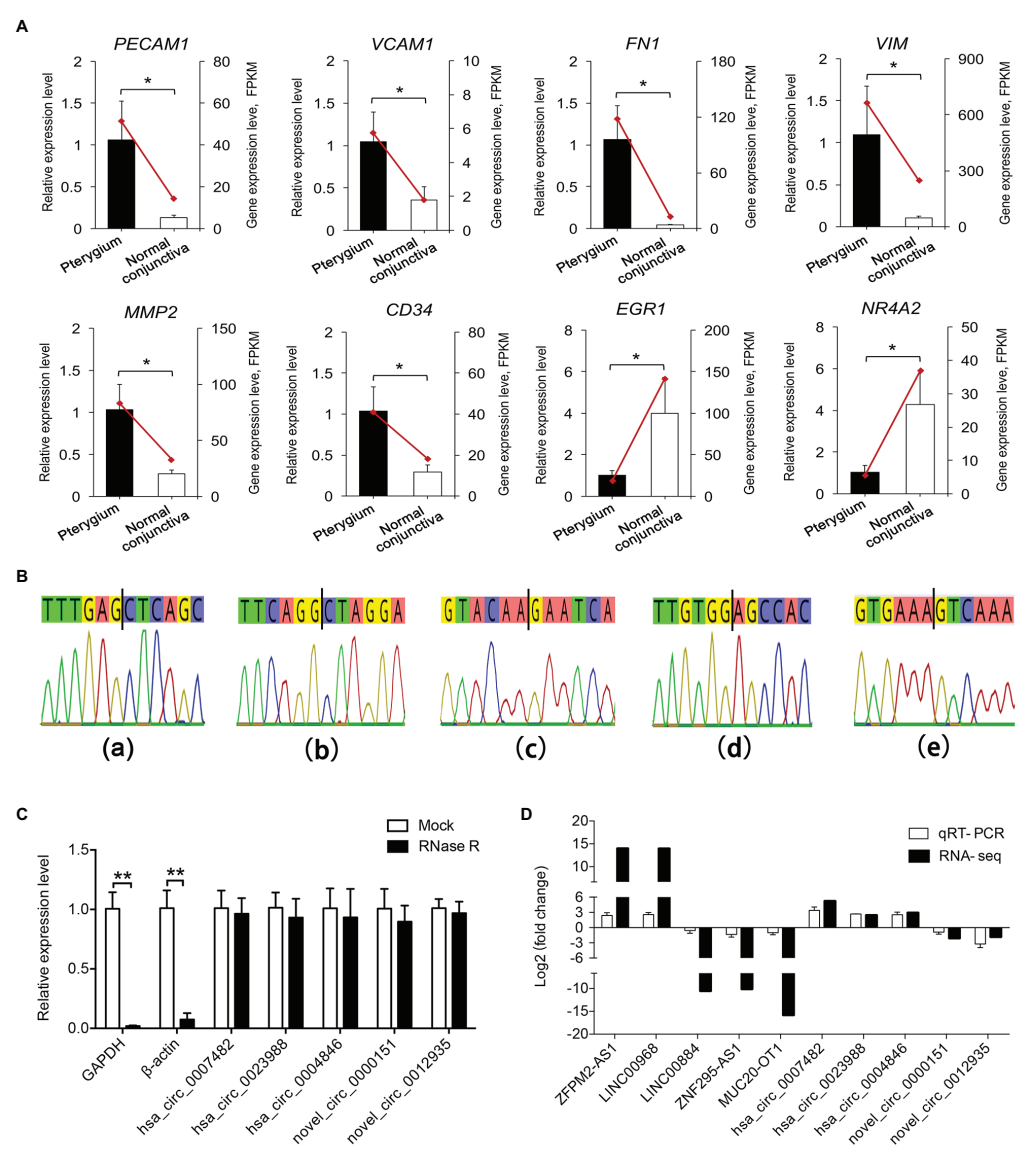
Validation of the DE genes. **(A)** Validation of the expression of DE mRNAs by qRT-PCR. Data from the quantitative real-time PCR (qRT-PCR) are shown as columns and are placed on the left *Y*-axis, while the data from RNA-seq are shown as lines and are placed on the right *Y*-axis. **(B)** Sanger sequencing-based validation for the back-splice site sequence of circRNA. Figures with small letters **(a–e)** represent five circRNAs in the following order: hsa_circ_0007482, hsa_circ_0023988, hsa_circ_0004846, novel_circ_0000151, and novel_circ_0012935, respectively. **(C)** RNase R digestion assays were performed to evaluate the stability of circRNAs. The *X*-axis indicates circRNAs, and the *Y*-axis indicates the relative expression levels of glyceraldehyde-3-phosphate dehydrogenase (GAPDH), β-actin, and circRNAs. **(D)** Validation of the expression of DE lncRNAs and circRNAs between pterygium vs. normal conjunctiva by qRT-PCR. Data are represented as mean ± SD, *n* = 3 per group. ^*^p < 0.05; ^**^p < 0.01.

To validate the RNA-seq data, the expression levels of eight mRNAs (*PECAM1*, *VCAM1*, *FN1*, *VIM*, *MMP2*, *CD34*, *EGR1*, and *NR4A2*) related to blood vessel morphogenesis, focal adhesion, and EMT progression, and five co-expressed lncRNAs (*ZFPM2-AS1*, *LINC00968*, *LINC00884*, *ZNF295-AS1*, and *MUC20-OT1*), were detected using qRT-PCR (Figures 8A,D). These genes showed considerably similar expression trends based on qRT-PCR results and RNA-seq data.

Five circRNAs (*hsa_circ_0007482*, *hsa_circ_0023988*, *hsa_circ_0004846*, *novel_circ_0000151*, and *novel_circ_0012935*) were selected to explore their splice sites according to Sanger sequencing results, and the RNase R digestion assay was used to assess the stability of circRNAs. The results of Sanger sequencing showed that the splice sites were consistent with those predicted by the software (Figure 8B). Furthermore, RNase R digestion assay results indicated that the five circRNAs were resistant to RNase R digestion compared to linear GAPDH and β-actin RNAs (Figure 8C). qRT-PCR results showed that the trends of five circRNAs expression were consistent with that of RNA-seq results (Figure 8D).

## Discussion

Pterygium is a common conjunctival degenerative disease, and pterygium excision with conjunctival autograft is the recommended treatment to reduce recurrence (Chu et al., 2020). Compared with the normal conjunctiva, pterygium possesses more vascular and fibrous tissues, and the morphology of these vessels suggests the existence of active angiogenesis in the upper subcutaneous connective tissue (Livezeanu et al., 2011; Engelsvold et al., 2013). Previous studies have performed the mRNA, miRNA, and lncRNA expression comparisons of pterygium and conjunctiva tissues *via* microarray or high-throughput sequencing technology (Liu et al., 2016, 2017a; He et al., 2019; Yue and Gao, 2019; Chen et al., 2020). However, studies on lncRNA and circRNA in pterygium development based on multiple types of RNA interaction analysis are limited. In this study, we first determined the expression profiles of lncRNA and circRNA in pterygium and conjunctiva by RNA-seq, and identified the biological processes and pathways related to the formation of pterygium. Abundant mRNAs, lncRNAs, and circRNAs that might be important in the pathogenesis of pterygium were identified through bioinformatics analysis and biochemical analysis.

Based on strict screening conditions, 579 DE mRNAs, 275 DE lncRNAs, and 21 DE circRNAs were obtained in this study. Functional enrichment analysis revealed that upregulated mRNAs were mainly related to extracellular matrix organization, blood vessel morphogenesis, and focal adhesion, while the downregulated mRNAs were mainly involved in cell death, which were consistent with the results of previous studies (He et al., 2019; Chen et al., 2020). Furthermore, the two most significant modules and 10 hub genes, including *FN1*, *MMP2*, *PECAM1*, *VWF*, *ENG*, *CXCL12*, *EGF*, *CD34*, *VCAM1*, and *CDH5*, were identified by PPI network analysis. Coincidently, several of the above mRNAs including *FN1*, *MMP2*, *VWF*, and *CXCL12* were identified as the hub genes associated with pterygium development in previous studies (He et al., 2019; Yue and Gao, 2019; Chen et al., 2020). Moreover, we validated several of these genes using qRT-PCR, and compared with RNA-seq data, similar expression trends were observed. These results suggest that angiogenesis and the changes in the extracellular matrix are involved in the formation of pterygium, which is consistent with the morphological characteristics of pterygium (Engelsvold et al., 2013).

Angiogenesis is a physiological process in human development and an important pathological mechanism in the development of diseases such as cancer, obesity, and ocular disease (Cristofanilli et al., 2002; Nijhawans et al., 2020; Scimone et al., 2020). Neovascularization and fibrous connective tissue are the main components of the pterygium, and an imbalance occurring between pro- and anti-angiogenic factors results in abnormal vascular proliferation (Livezeanu et al., 2011; Tonthat and Di Girolamo, 2014). Compared with the conjunctiva, several key factors related to angiogenesis, such as *PECAM1*, *CD34*, *VWF*, and *ENG*, were highly expressed in the pterygium. *PECAM1*, an important endothelial cell marker, is mainly expressed in vascular endothelial cells (Murray et al., 2019; Tan et al., 2019). Consistent with our results, immunostaining of *PECAM1* revealed much richer vascularization in pterygium (Aspiotis et al., 2007; Livezeanu et al., 2011). Additionally, *CD34*, *VWF*, and *ENG*, endothelial cell surface markers, and their positive microvascular counts were markedly higher in pterygium than those in conjunctival tissue (Marcovich et al., 2002; Lee et al., 2007; Zhang et al., 2011). During angiogenesis, vascular endothelial cells must migrate through the extracellular matrix, and this process is strictly controlled by the action of cell adhesion molecules (Bischoff, 1997). *VCAM-1* is a member of the immunoglobulin superfamily and an important cell adhesion molecule, the positive rate of which in pterygium is higher than that observed in conjunctiva (Tekelioglu et al., 2006). Moreover, *CDH5* and *CDH6*, two important epithelial cell adhesion factors, were highly expressed in pterygium tissues investigated in this study. These factors promote neovascularization, provide nutrition for the proliferation of pterygium, and accelerate pterygium growth.

Degradation and reconstruction of the extracellular matrix are key steps for angiogenesis, and these steps are controlled by the action of the members of the matrix metalloproteinases (MMPs) family (Sang, 1998; Reid and Dushku, 2010). Vascular endothelial cells secrete a variety of MMPs, which can degrade the extracellular matrix individually or in concert, and promote angiogenesis (Sang, 1998). In this study, multiple types of MMPs (*MMP2*, *MMP3*, *MMP8*, *MMP9*, *MMP11*, *MMP16*, and *MMP28*) were highly expressed in pterygium. The expression of MMP1, an important factor in corneal collagen degradation, in pterygium head fibroblasts is higher than that in the body and conjunctiva, which creates conditions for degradation of the exposed elastic layer by MMP2 (Li et al., 2001). *MMP2* and *MMP9* encode two different forms of gelatinase, are produced after the pterygium cells invading over Bowman’s layer, which contribute to the dissolution of Bowman’s layer (Dushku et al., 2001). Previous studies have shown that *MMP2* and *MMP9* were expressed in advanced-stage pterygium, but they were not detected in early-stage pterygium tissues and fibroblasts (Yang et al., 2009).

Extracellular matrix remodeling enables pterygium-mediated invasion of the cornea, while EMT is another important mechanism responsible for the promotion of the migration and invasion of pterygium epithelial cells (Oh et al., 2014; Yue and Gao, 2019). Kato et al. (2007) found that newly synthesized collagen fibrils accumulated between the epithelium and Bowman’s layer at the pterygium head. *FN1*, an important factor involved in cell adhesion and migration processes, was enriched in the PPI network in this study (Cai et al., 2017). Transforming growth factor-β is a master switch for myofibroblasts, which can induce the synthesis of *FN1* and *α-SMA* in primary human pterygium fibroblasts and primary human conjunctival fibroblasts, and the expression levels of TGF-β2 and TGF-β3 were both upregulated in this study (Di Girolamo et al., 2004; Chen et al., 2019). *VIM* is a major cytoskeletal component of mesenchymal cells. Expression of *VIM* and *α-SMA* was detected in epithelial keratins from pterygium tissue but not in normal corneal epithelium (Kato et al., 2007). Upregulation of these EMT markers is an important mechanism of pterygium fibrosis and the main cause of the high recurrence rate of pterygium.

Previous reports have shown that multiple lncRNAs were DE in pterygium compared with normal conjunctiva, but the function of lncRNAs in the pathogenesis of pterygium has not been studied extensively (Liu et al., 2016). The potential functions of important lncRNAs were predicted based on the mRNA-lncRNA co-expression network created in this study. The results showed that these DE lncRNAs were mainly involved in angiogenesis and focal adhesion, which might play a key role in the pathogenesis of pterygium. Through the analysis of 10 hub mRNAs and their co-expressed lncRNAs, a considerable number of key lncRNAs that might be involved in the pathogenesis of pterygium were screened. *MIR4435-2HG* was co-expressed with *FN1* and *MMP2* and was highly expressed in pterygium. It can promote the proliferation and metastasis of various cancers by upregulating TGFβ-1 expression or by promoting the occurrence of EMT (Yang et al., 2019; Zhang et al., 2020). Moreover, *LINC00968*, which is co-expressed with *ENG* and *VCAM1*, can promote the proliferation and migration of endothelial cells and accelerate the proliferation and fibrosis in diabetic nephropathy (Li et al., 2018b; Wang et al., 2018). However, another study showed that *LINC00968* could inhibit breast cancer cell proliferation, migration, and angiogenesis (Sun et al., 2019). Additionally, *ST3GAL6-AS1* was co-expressed with a variety of mRNAs, including *PECAM1*, *VCAM1*, *CXCL12*, *CD34*, *CDH5*, and *VWF*, and was associated with colorectal cancer progression (Hu et al., 2019). In-depth functional analysis of these lncRNAs would enable the identification of key genes regulating the formation of pterygium, which may provide new insights for the development of strategies for the treatment of pterygium.

In addition to lncRNAs, circRNAs play an important role in the pathogenesis of pterygium (Li et al., 2018a). By GO functional analysis of parental genes of circRNAs, we first revealed that the upregulated circRNAs were mainly related to protein kinase B signaling, epithelial tube formation, and positive regulation of apoptotic process, while downregulated circRNAs were associated with epithelial cell development, angiogenesis, and positive regulation of mesenchymal cell proliferation. Interestingly, few DE circRNAs have been reported to be involved in tumor progression and proliferative diseases. For instance, *hsa_circ_0007482* expression was upregulated in pterygium in this study, and its expression level in keloid tissue was upregulated compared with that in normal skin tissue (Shi et al., 2019). Furthermore, *hsa_circ_001730* expression was upregulated in pterygium and was reported to promote the proliferation and invasion of glioma cells (Lu et al., 2019). These DE circRNAs may be involved in the regulation of abnormal proliferation and postoperative recurrence of pterygium, and it is necessary to further explore their molecular function.

Furthermore, several studies have shown that UV radiation and ion channel dysfunction are also involved in the pathogenesis of pterygium (Cimpean et al., 2013; Garreis et al., 2016; Liu et al., 2017a). Recent reports have revealed that DNA damage and mutations in ion channel-related genes are also closely related to the occurrence and progression of certain ophthalmic diseases, including retinal degeneration and retinal dystrophy (Donato et al., 2020a,b). In this study, the genes related to response to oxidative stress, including *FOS*, *JUN*, and *DUSP1*, were significantly downregulated, and the genes involved in ion transmembrane transport, such as *CALM1*, *CALM2*, and *FXYD4*, were also significantly downregulated. Therefore, oxidative stress injury caused by UV exposure and ion channel dysfunction resulting in abnormal cell proliferation and apoptosis, which may also be important factors in the occurrence of pterygium. In general, pterygium is a multifactorial disease, and further studies are warranted to elucidate its pathogenesis in the future.

## Conclusion

In this study, we revealed that several mRNAs, lncRNAs, and circRNAs might participate in pterygium formation by regulating cell adhesion, EMT, and angiogenesis. Through the construction of the PPI network and mRNA-lncRNA co-expression networks, we screened for important mRNAs and lncRNAs that might be related to the pathogenesis of pterygium. This study provides a comprehensive differential expression profile of different types of RNA in pterygium, which helps us to explore the molecular pathomechanism of pterygium.

## Data Availability Statement

RNA-seq data were submitted to NCBI SRA (accession number: PRJNA669964).

## Ethics Statement

The studies involving human participants were reviewed and approved by the Ethics Committee of the Shenzhen Eye Hospital. The patients/participants provided their written informed consent to participate in this study.

## Author Contributions

XL, JZ, XhL, and JW designed the experiment and wrote and modified the manuscript. XL, DN, and JT performed the experiment and bioinformatics analysis. KZ, HH, LS, and LP collected the samples. All authors contributed to the article and approved the submitted version.

### Conflict of Interest

The authors declare that the research was conducted in the absence of any commercial or financial relationships that could be construed as a potential conflict of interest.

## Footnotes

^1^www.omicshare.com/tools

^2^https://string-db.org/

## Supplementary Material

Supplementary Figure S1 Principal component analysis (PCA) of mRNA, lncRNA, and circRNA expression profiles. PCA of mRNA **(A)**, lncRNA **(B)**, and circRNA expression profiles **(C)** identified based on the analysis of six samples with three biological replicates.
