# Supplementary material for: Comparative Transcriptomic Analysis to Identify the Important Coding and Non-coding RNAs Involved in the Pathogenesis of Pterygium
Source: Front Genet. 2021 Mar 15;12:646550. doi: 10.3389/fgene.2021.646550 (PMC8005612; doi:10.3389/fgene.2021.646550)
Supplement: Supplementary Figure S1 — Principal component analysis (PCA) of mRNA, lncRNA, and circRNA expression profiles. PCA of mRNA (A), lncRNA (B), and circRNA expression profiles (C) identified based on the analysis of six samples with three biological replicates. [file Data_Sheet_1.zip › Supplementary_Material/Figure S1 Principal component analysis (PCA) of mRNA, lncRNA, and circRNA expression profile.pdf]

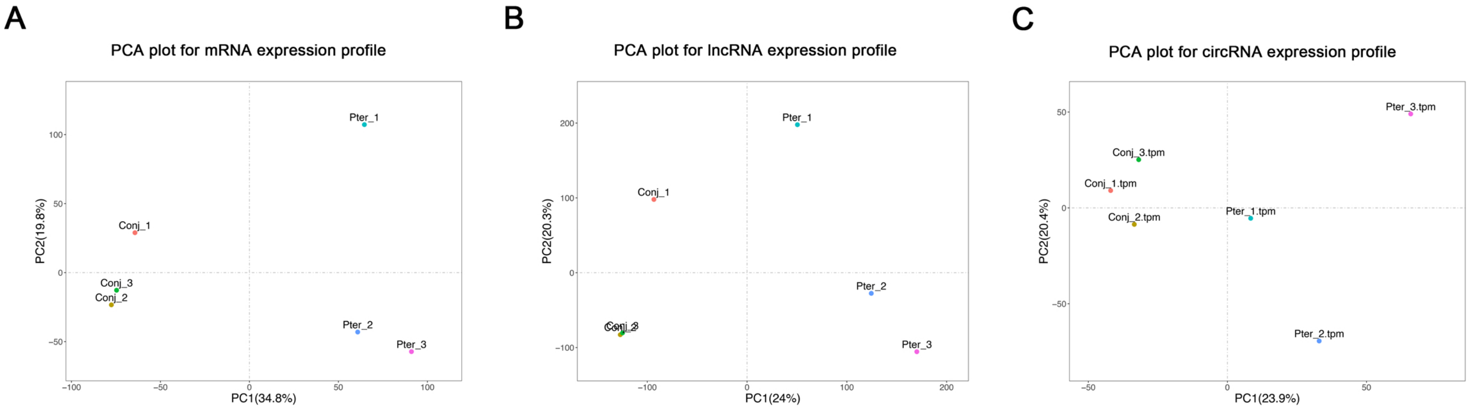
A
PCA plot for mRNA expression profile
PC1(34.8%)
PC2(19.8%)
Pter_1
Conj_1
Conj_3
Conj_2
Pter_2
Pter_3
B
PCA plot for lncRNA expression profile
PC1(24%)
PC2(20.3%)
Pter_1
Conj_1
Pter_2
Pter_3
C
PCA plot for circRNA expression profile
PC1(23.9%)
PC2(20.4%)
Pter_3.tpm
Conj_3.tpm
Conj_1.tpm
Pter_1.tpm
Conj_2.tpm
Pter_2.tpm
